# Level and factors of support for the Living with the Virus policy in a Chinese adult general population: a mediation analysis via positive and negative attitudes toward the policy

**DOI:** 10.3389/fpubh.2024.1286596

**Published:** 2024-01-29

**Authors:** Joseph T. F. Lau, Joyce Hoi-Yuk Ng, Robin Goodwin, Tarandeep S. Kang, Vivian W. I. Fong, Mason M. C. Lau, Yanqiu Yu

**Affiliations:** ^1^Public Mental Health Center, School of Mental Health, Wenzhou Medical University, Wenzhou, China; ^2^Zhejiang Provincial Clinical Research Center for Mental Disorders, The Affiliated Wenzhou Kangning Hospital, Wenzhou Medical University, Wenzhou, China; ^3^Center for Health Behaviors Research, Jockey Club School of Public Health and Primary Care, The Chinese University of Hong Kong, Sha Tin, Hong Kong SAR, China; ^4^Department of Psychology, University of Warwick, Coventry, United Kingdom; ^5^Department of Preventive Medicine and Health Education, School of Public Health, Fudan University, Shanghai, China

**Keywords:** Living with the Virus, COVID-19, policy support, cognition, public attitude

## Abstract

**Background:**

This study investigated the public’s support for the Living with the Virus (LWV) policy, its associated factors, and related mediations at a time when more countries were considering adopting the LWV policy amid the COVID-19 pandemic.

**Methods:**

A random, population-based telephone survey was conducted among 500 Chinese adults in Hong Kong during March/April 2022.

**Results:**

39.6% of the participants were supportive/strongly supportive of the LWV policy. Perceived efficacy of the control measures was negatively associated with the support and was partially mediated via the perception that the policy would greatly improve the economy/daily life of the policy. Perceived physical harms of the Omicron variant was negatively associated with the support and was fully mediated via perceived negative impacts of the policy. 26.2%/54.6% believed that the policy would improve the economy/daily life greatly; about 40% perceived negative impacts on deaths and the medical system due to the policy. COVID-19 ever infection did not significantly moderate the studied associations.

**Conclusion:**

The public was split regarding the support for the LWV policy and whether it would cause better economy/daily life, unnecessary deaths, and the collapse of the medical system. Health communication is needed in shifting toward the LWV policy.

## 1 Introduction

The world shared common responses to the COVID-19 pandemic, including national vaccination programs (1–3), compulsory mask use in public areas (4), travel restrictions (e.g., testing, quarantines, and bans for inbound/outbound travels), social distancing measures ranging from milder (e.g., restrictions of gathering size, closure of venues, and working from home) to stricter (e.g., class suspension, banning social visits, and lockdowns) measures, although the extent, duration, and compliance of these responses varied across countries and regions. Many governments provided free COVID-19 testing to the public, including large-scale community-based testing (e.g., China) (5, 6). Such preventive measures involved extremely high social, psychological, and economic costs (7, 8). A balance between control versus costs was difficult to achieve and often politicized (9).

China initially adopted the “Dynamic Clearance to Zero” (DCZ) policy, which involved stringent measures terminating transmission chains through controlling international and domestic travel, lockdowns, and universal COVID-19 testing. The policy intended to buy time to increase the vaccination rate and the development of effective treatments, and it kept the entire country operating normally for 2 years. Other countries (e.g., Australia) had also adopted the DCZ policy and had been able to maintain a zero-case state for some periods. However, the extremely infectious Omicron variant posed new challenges to the world due to the extremely large number of new COVID-19 cases and the high economic and psychosocial costs related to the control measures.

At the opposite end of the spectrum, the Living with the Virus (LWV) policy referred to lifting almost all COVID-19 restrictions, with rationales that COVID-19 would become an endemic when substantial community immunity had been established through natural and vaccination-induced immunity. The U.S. and the U.K. were close to implementing the LWV policy at the beginning of the pandemic but soon made a reversion when the number of cases and deaths soared exponentially. Sweden initially and truly adopted the LWV policy in February 2022 (10), and then more countries followed suit, including Singapore, European countries (e.g., the Netherlands, Switzerland, Austria, Germany, Poland, Iceland, Denmark, Norway), Australia, and New Zealand; Thailand, Japan, South Korea, and Indonesia dropped quarantines for travelers. Such policy changes were embedded in epidemiological updates (e.g., high infectivity and milder nature of the Omicron variant (11), high national vaccination rates, decline in the global new COVID-19 cases (3), and the availability of promising treatments) and social trends (e.g., global fatigue to strict restrictions and severely damaged economies (12)). More countries were, however, observing and considering their preparedness (e.g., vaccination rates) for and potential impacts of adopting the LWV policy, as COVID-19 remained a pandemic as of May 10th, 2022 (3).

Prior to and during the time of the present study, there was heated debate on the DCZ versus the LWV policy when more and more countries were considering the policy shift (13, 14). The general public’s support for the policy would be impactful. First, public attitude (support or rejection) might facilitate health communication, influencing policymakers to prioritize, enact, or modify the policy (15). Second, public support could build up a norm and to some extent contribute to social cohesion, leading to stronger engagement and compliance with the policy (16). Third, public support might enhance the trust in the government, which may further increase the acceptance and adherence to the policy (15). To facilitate policymaking, health communication, and compliance with the policy, it is hence warranted to understand the level of support for the LWV policy and associated factors in the public. Numerous behavioral change theories (e.g., Health Belief Model, Theory of Planned Behaviors, and Protection Motivation Theory) highlight the importance of perceptions in determining the attitude towards and compliance with health policies (17–19). Empirically and specifically, the literature has documented that those favorable perceptions related to COVID-19 policies and control measures (e.g., perceived efficacy of social distancing and COVID-19 vaccination) were positively associated with the support for these policies and the adoption of related measures (9, 20, 21). Accordingly, the public’s perceptions of the LWV policy might be associated with their attitude towards the LWV policy and the subsequent policy compliance. However, to our knowledge, no studies have investigated perceptions specific to the LWV policy; our literature search located only two studies that investigated the general emotional and cognitive factors of the public’s support for the LWV policy, including self-efficacy, resilient coping, emotional distress, and illness perceptions of COVID-19 (22, 23).

This study aimed to fill out the knowledge gap on the associations between perceptions specific to the LWV policy and the support for the policy. During the time of this study, although COVID-19 might become an endemic that would no longer require strong control measures, there were uncertainties. For instance, the benefits of the LWV policy included an opportunity to return to normal life and a potential chance to control the pandemic sooner via natural immunity. In contrast, there were worries about potential surges of infections/deaths and the emergence of highly infectious and severe variants (3). Such positive and negative perceptions might jointly determine the general public’s support for the policy, as previous literature reported that positive and negative outcome expectancies of a COVID-19-related health policy (e.g., COVID-19 vaccination and social distancing) were associated with the support for the policy (9, 20, 21). Other perceptions might also count. As the Omicron variant had higher infectivity but lower severity than the original/Delta COVID-19 strains, the present study contended that those who perceived Omicron as being less severe would have less negative attitude and thus a higher level of support for the LWV policy. Another potential factor is the perceived efficacy of the existing COVID-19 control measures. Those who perceive the high efficacy of the control measures might see the removal of such measures by the LWV policy as being less beneficial; they might hence be more likely to perceive negative effects of the LWV policy, such as causing unnecessary deaths and the collapse of the medical system, and hence be less likely to support the LWV policy. Such associations were assessed by the present study for the first time.

Given the background, the present study investigated the level of support for the LWV policy in the Chinese adult general population in Hong Kong. Factors of support for the LWV policy were investigated: (a) perceived efficacy of various COVID-19 control measures, (b) perceived physical harms of the Omicron variant, (c) two types of perceived benefits of the LWV policy, and (d) perceived negative impacts of the LWV policy. It was hypothesized that the correlations would be negative between (a)/(b)/(d) and the support and positive involving (c). The moderation hypothesis that the above associations would differ according to COVID-19 ever infection was tested. It also tested whether the association between perceived efficacy of COVID-19 control measures and the support would be mediated via perceived benefits/negative impacts of the LWV policy, and whether the association between perceived physical harms of the Omicron variant and the support would be mediated via perceived negative impacts of the policy.

## 2 Methods

### 2.1 Data collection

A random telephone survey was conducted in Chinese adults aged ≥18 years from March 7th to April 19th, 2022, i.e., during the fifth-wave COVID-19 outbreak in Hong Kong. The city had been adopting the DCZ policy since the early phase of the pandemic. In March 2022 when the survey was conducted, local control measures included quarantine for all inbound travelers, patients, and close contacts, curbing incoming flights, universal free rapid antigen test (RAT), isolating residents of housing blocks where infections were found, working from home, school suspension, closure of catering and entertainment venues, compulsory facemask use in public areas, and restricting non-vaccinated people to enter public venues. People were fined and put into jail for violations. There were nil new COVID-19 cases during a 3-month period (October 2021 to early January 2022) (24). A massive fifth-wave outbreak then of 192,765 cases and 9,115 deaths out of a population of 7.3 million occurred from February 2022 to late April 2022 (24), which peaked at about 56,000 new infections per day (March 3rd, 2022). The number of cases dropped to less than 500/day after April 24th, 2022. As of April 1st, 2022, the 2-dose vaccination rate was 78.7% (24). Since late April 2022, the government started loosening some but not all social distancing policies (e.g., reopening of restaurants). Thus, the city seemed to be shifting from the DCZ policy to a hybrid DCZ and LWV policy.

A total of 480,000 household telephone numbers were randomly drawn from the updated landline telephone directories in Hong Kong. To cover unlisted telephone numbers, three additional numbers were generated by randomizing the last two digits of each randomly selected number. The two sets of numbers were merged to form the sampling frame. Invalid numbers (e.g., commercial numbers and fax numbers) were replaced by additional random numbers. Interviews were made from 5 pm to 10 pm (10 to 15 min) by experienced interviewers to avoid over-sampling non-working individuals. The household member whose birthday was closest to the interview date was invited to join the study. Unanswered telephone calls were given at least three attempts before being classified as invalid. Unavailable eligible participants were contacted again by appointment. No incentives were given to the participants. Verbal informed consent was obtained from the participants. Ethics approval was obtained from the Survey and Behavioral Research Ethics Committee of the corresponding author’s affiliated institution (No. SBRE-21-0555A). A total of 957 valid contacts were made; 500 interviews were completed (response rate = 52.2%).

### 2.2 Measures

An expert panel was set up to develop the structured questionnaire; the panel comprised one behavioral scientist, one public health expert, and two health psychologists, based on a comprehensive literature review of COVID-19-related perceptions and policies; the face validity and content validity of the items were determined by the experts by consensus. A pilot survey was then conducted among ten adults in Hong Kong to assess the clarity, readability, and length of the questionnaire. With their feedback, the panel finalized the questionnaire.

#### 2.2.1 Background characteristics

Information about age, sex, educational level, marital status, employment status, and chronic disease status (e.g., hypertension, diabetes, chronic pulmonary diseases, heart diseases, cerebrovascular diseases, dementia, liver diseases, tumors; yes or no response options), and COVID-19 ever infection status (based on the testing results of Nucleic Acid Amplification Test [NAAT] or RAT) was collected.

#### 2.2.2 Support for the LWV policy

The item was: “Based on the current local COVID-19 situation, to what extent do you support implementing the LWV policy in Hong Kong, which means cancelation of the COVID-19 policies regarding (a) social distancing, (b) compulsory facemask use, (c) free and compulsory COVID-19 testing, (d) travel restrictions, and (e) quarantine?” The five-point response options were recoded into a binary dependent variable [1 = supportive (supportive/strongly supportive) versus 0 = unsupportive (neutral/unsupportive/strongly unsupportive)].

#### 2.2.3 Perceived efficacy of the existing COVID-19 control measures

A 6-item scale assessed perceived efficacy of existing local measures, including (1) three doses of vaccination, (2) compulsory facemask use in public areas, (3) social distancing, (4) a 90% vaccination rate, (5) compulsory COVID-19 testing for all residents of housing blocks having a few COVID-19 cases, and (6) overall COVID-19 control policy in Hong Kong (1 = extremely low to 5 = extremely high; Cronbach’s alpha = 0.8).

#### 2.2.4 Perceived physical harms of the omicron variant

A 2-item scale assessed the levels of agreement with the two statements: “The short-term physical harms of the Omicron variant are severe” and “Omicron may cause severe long-term physical harms” (1 = strongly disagree to 5 = strongly agree; Cronbach’s alpha = 0.7).

#### 2.2.5 Perceived benefits and negative impacts of the LWV policy

Two items assessed the levels of agreement with two statements: “The LWV policy can contribute to the effective control of the COVID-19 pandemic in Hong Kong” and “The LWV policy can greatly improve the economy and people’s daily life in Hong Kong.” Negative impacts were assessed by two statements: “The LWV policy would cause many unnecessary deaths in Hong Kong” and “The LWV policy would lead to the collapse of the medical system in Hong Kong” (1 = strongly disagree to 5 = strongly agree).

### 2.3 Statistical analysis

Pearson correlation coefficients were derived to test the interrelationships among the perception variables. Univariable and multivariable logistic regression analyzes tested the significance and directions of the individual associations between the perception variables and the support for the LWV policy. The moderation effects of COVID-19 ever infection between the perception variables and the support for the LWV policy were tested by using hierarchical logistic regression analyzes. Two models were fit for each perception variable: (1) the main-effect-only model involved one perception variable (e.g., perceived efficacy of the existing COVID-19 control measures) and the potential moderator (i.e., COVID-19 ever infection), and (2) the second model added the interaction term (e.g., perceived efficacy of the existing COVID-19 control measures × COVID-19 ever infection) to the first model. Six sets of the above two models were fit in total. Path analyzes, using the Weighted Least Square Mean and Variance Adjusted estimator, were conducted to test the mediations (1) between perceived efficacy of the existing COVID-19 control measures and support for the LWV policy via perceived benefits and negative impacts of the LWV policy, and (2) between perceived physical harms of the Omicron variant and support for the LWV policy via perceived negative impacts of the LWV policy. Path analyzes were conducted by using Mplus 7.0. The other statistical analyzes were conducted by using SPSS 23.0. All multivariable logistic regression, mediation, and moderation analyzes were adjusted for background variables.

## 3 Results

### 3.1 Descriptive statistics

Socio-demographic information is presented in Table 1. Two-thirds of the participants were female (67.0%) and currently married (68.8%); about one-third aged >60 years (33.4%). About one-fifth to a quarter had received education of college or above (24.6%), had a full-time job (41.6%), and had at least one of the listed chronic diseases (35.8%). About one-sixth (16.8%) self-reported COVID-19 ever infection. About 40% (39.6%) were supportive/strongly supportive of the LWV policy in Hong Kong; 26.2% agreed/strongly agreed that the LWV policy would contribute to the control of the pandemic; 54.6% agreed/strongly agreed that it would greatly improve the economy and daily life; 42.0 and 50.4% believed that the policy would cause many unnecessary deaths and the collapse of the local medical system, respectively.

**Table 1:** Participant characteristics (*n* = 500).

	*n*	%
*Socio-demographics*		
Sex		
Female	337	67.0
Male	165	33.0
Age group (years)		
18–30	71	14.2
31–60	262	52.4
>60	167	33.4
Educational level		
Below college	365	73.0
College or above	123	24.6
Missing data	12	2.4
Marital status		
Others	151	30.2
Married	344	68.8
Missing data	5	1.0
Employment status		
Full-time	208	41.6
Part-time	41	8.2
Retired	114	22.8
Under-employed	27	5.4
Homemaker	94	18.8
Others	16	3.2
Chronic disease status		
No/unknown	321	64.2
Yes	179	35.8
*COVID-19 ever infection*		
No	394	78.8
Yes	84	16.8
Do not know	16	3.2
Refused to answer	6	1.2
*Support for the LWV policy*		
Strongly unsupportive	70	14.0
Unsupportive	65	13.0
Neutral	167	33.4
Supportive	130	26.0
Strongly supportive	68	13.6
*Perceived benefits of the LWV policy*		
Contribution to the control of the pandemic		
Strongly disagree	66	13.2
Disagree	88	17.6
Neutral	215	43.0
Agree	113	22.6
Strongly agree	18	3.6
Great improvement in the economy and daily life		
Strongly disagree	23	4.6
Disagree	48	9.6
Neutral	456	31.2
Agree	216	43.2
Strongly agree	57	11.4
*Perceived negative impacts of the LWV policy*		
Causing many unnecessary deaths		
Strongly disagree	17	3.4
Disagree	88	17.6
Neutral	185	37.0
Agree	129	25.8
Strongly agree	81	16.2
Causing the collapse of the local medical system		
Strongly disagree	14	2.8
Disagree	77	15.4
Neutral	157	31.4
Agree	146	29.2
Strongly agree	106	21.2

LWV, Living with the virus.

### 3.2 Correlations

All perception factors were significantly correlated with each other, except the one between perceived efficacy of the existing COVID-19 control measures and perceived contribution of the LWV policy to the control of the pandemic (see Supplementary Table S1). In general, perceived efficacy of the existing COVID-19 control measures and perceived physical harms of the Omicron variant were positively correlated with the items of perceived negative impacts of the policy, and negatively correlated with the items of perceived benefits of the policy. The items of perceived benefits of the LWV policy were negatively correlated with those of perceived negative impacts of the policy. The absolute Pearson correlation coefficients ranged from 0.20 to 0.71 (*p* < 0.05).

### 3.3 Factors of support for the LWV policy

Older age, being currently married, and not having a full-time job were negatively associated with the support for the LWV policy, while college or above education and COVID-19 ever infection were positively associated with the support. Adjusted for these socio-demographics, the multivariable logistic regression analyzes found that perceived efficacy of the existing COVID-19 control measures (ORa = 0.60; 95% CI: 0.44, 0.81) and perceived physical harms of the Omicron variant (ORa = 0.74; 95% CI: 0.58, 0.94) were negatively associated with the support for the LWV policy. The two types of perceived benefits of the LWV policy (i.e., perceived contribution to the control of the pandemic and great improvement in the economy and daily life) were positively associated with the support for the LWV policy [ORa = 2.29 (95% CI: 1.83, 2.88) and 3.07 (95% CI: 2.31, 4.07), respectively]. The two types of perceived negative impacts of the LWV policy (i.e., causing unnecessary deaths and the collapse of the local medical system) were negatively associated with the support for the LWV policy [ORa = 0.57 (95% CI: 0.46, 0.69) and 0.64 (95% CI: 0.53, 0.78), respectively]. The crude odds ratios (ORc) were similar to the corresponding ORa (Table 2). As shown in Supplementary Table S2, none of the associations were significantly moderated by COVID-19 ever infection.

**Table 2:** Factors of support for the LWV policy.

	ORc (95% CI)	ORa (95% CI)
Perceived efficacy of the existing COVID-19 control measures	0.55 (0.42, 0.72)***	0.60 (0.44, 0.81)**
Perceived physical harms of the Omicron variant	0.70 (0.56, 0.88)**	0.74 (0.58, 0.94)*
*Perceived benefits of the LWV policy*		
Contribution to the control of the pandemic	2.25 (1.81, 2.78)***	2.29 (1.83, 2.88)***
Great improvement in the economy and daily life	2.76 (2.14, 3.55)***	3.07 (2.31, 4.07)***
*Perceived negative impacts of the LWV policy*		
Causing many unnecessary deaths	0.56 (0.47, 0.68)***	0.57 (0.46, 0.69)***
Causing the collapse in local medical system	0.61 (0.52, 0.74)***	0.64 (0.53, 0.78)***

LWV, Living with the virus; ORc, Crude odds ratio; CI, Confidence interval; ORa, Adjusted odds ratio. *p < 0.05; **p < 0.01; ***p < 0.001. The adjusted models were adjusted for socio-demographics, including sex, age, educational level, marital status, chronic disease status, and employment status.

### 3.4 Path analysis

**Figure 1:**
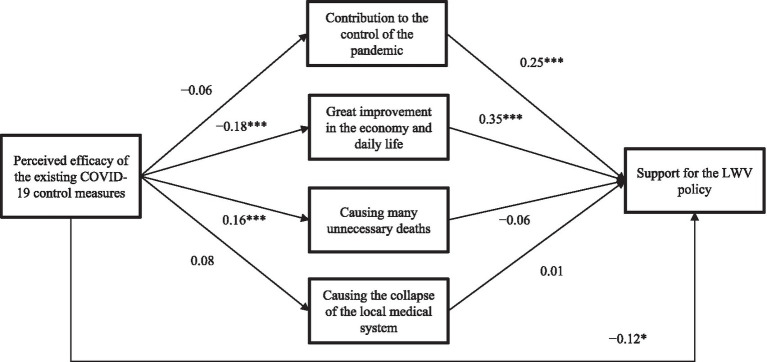
Path analysis of the mediation effect of perceived benefits/negative impacts of the LWV policy between perceived efficacy and support for the LWV policy (LWV, Living with the virus; *, *p* < 0.05; ***, *p* < 0.001. Standardized coefficients were reported. The model was adjusted for socio-demographics. The correlations among the four variables of perceived benefits and negative impacts of the LWV policy were significant but not presented in the model for simplification).

Two significant indirect paths were found in Figure 1. (1) The indirect effect via perceived benefit of great improvement in the economy and daily life was statistically significant (*β* = −0.10; *p* = 0.001), i.e., perceived efficacy was negatively associated with this perceived benefit (*β* = −0.18; *p* < 0.001), which was in turn positively associated with the support for the LWV policy (*β* = 0.35; *p* < 0.001). (2) The direct effect (*β* = −0.12; *p* = 0.042) from perceived efficacy to the support for the LWV policy was statistically significant, indicating a partial mediation effect of perceived benefit of great improvement in the economy and daily life (mediation effect size = 30.5%). The other indirect paths via (a) perceived contribution to the control of COVID-19 (*β* = −0.02; *p* = 0.222), (b) perceived causing many unnecessary deaths (*β* = −0.02; *p* = 0.404), and (c) perceived collapse of the local medical system (*β* = 0.01; *p* = 0.951) were statistically non-significant.

**Figure 2:**
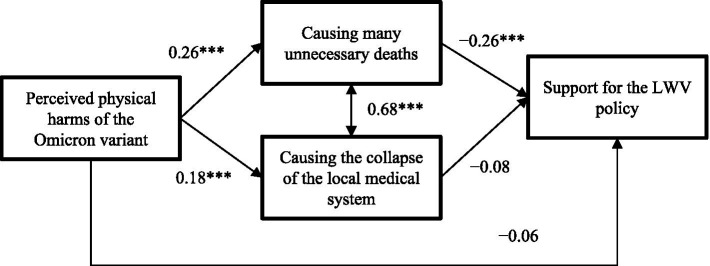
Path analysis of the mediation effect of perceived negative impacts of the LWV policy between perceived physical harms of the Omicron variant and support for the LWV policy (LWV, Living with the virus; ***, *p* < 0.001. Standardized coefficients were reported. The model was adjusted for the socio-demographics).

Figure 2 shows that perceived negative impacts of causing many unnecessary deaths fully mediated the association between perceived physical harms of the Omicron variant and the support for the LWV policy (*β* = −0.07; *p* < 0.001), i.e., perceived physical harms of the Omicron variant was positively associated with perceived negative impacts of the policy (*β* = 0.26; *p* < 0.001), which was in turn negatively associated with the support for the LWV policy (*β* = −0.26; *p* < 0.001). The indirect path via perceived collapse of the local medical system (*β* = −0.01; *p* = 0.315) and the direct path (*β* = −0.06; *p* = 0.252) from perceived physical harms of the Omicron variant to the support for the LWV policy were statistically non-significant.

## 4 Discussion

During the survey period when a severe outbreak occurred, the government strongly endorsed the DCZ policy and exercised strong control measures (25), synchronizing with the national COVID-19 policy (26). In contrast, about 40% of the general population supported the LWV policy, reflecting a substantial discrepancy between the working policy and public opinion. No international data was available for comparisons. Younger, better-educated, single, and employed people were more likely than others to support the LWV policy, possibly because their lives were more likely to be affected by the COVID-19 control measures. Individuals with COVID-19 ever infection were more supportive of the policy, possibly because this population developed mild symptoms and perceived adequate immunity.

About 17% of the participants self-reported having COVID-19 ever infection, which was comparable to the official data of 16% reported on April 19th, 2022 (27). However, the prevalence might have been underestimated, as many asymptomatic and mild cases might not perform COVID-19 testing or report to the government. A modeling study suggested that >60% of the Hong Kong general population might have contracted COVID-19 as of April 2022 (28). As the perception that the city had built up substantial community immunity through natural immunity and the high vaccination rate (about 90%) might prevail, together with the prevention fatigue, it was expected that the level of support for the LWV policy would rise in the near future.

This study investigated the levels of perceptions specific to a public health policy during a time when the policy shift was considered by many countries. Novel observations of how people perceived the positive and negative impacts of the LWV policy might facilitate health communication between the government and the public. Understanding the public’s concerns, misconceptions, and needs allows health authorities to tailor their communication strategies that would guide health behavior and reduce mental distress. In this study, the public remained uncertain about the policy’s benefits (e.g., contribution to the control of the pandemic and great improvement in the economy and life), and some were concerned about causing many unnecessary deaths and the collapse of the local medical system. Notably, such perceptions might change along with the epidemiological situations and the emergence of new vaccines/treatments, as indicated by the findings of numerous studies (29–31). Clear and accurate information should hence be updated, and misinformation should be corrected promptly by health authorities.

This study also investigated whether and how the above perceptions would affect public support for the policy. The significant associations between perceived benefits/negative impacts of the policy and the support corroborated previous findings that perceptions of a public health policy affected the attitude towards that policy (32, 33). It suggests that, if people understand and agree with the necessities of a policy or if they have fewer worries about the negative consequences of a policy, they might be more likely to support the policy, which might further result in greater acceptance, trust, and compliance with the policy.

In addition, the revealed mechanisms were novel in unraveling how people’s perceptions of policy would be affected by other COVID-19-related perceptions. Those perceiving severe short-term and long-term physical harms of the Omicron variant were less supportive of the LWV policy. As the LWV policy implied higher risks of COVID-19 infection; the consequences could be threatening if the Omicron infection was very harmful. The partial mediation of this association via perceived causing many unnecessary deaths and the collapse of the local medical system is understandable, as those perceiving harmful consequences of the Omicron variant might tend to believe that the LWV policy would have negative impacts and hence be unsupportive. The negative association between perceived efficacy of the existing COVID-19 control measures and the support was also significantly and partially mediated via perceived great improvement in the economy and daily life. Those with perceived efficacy of the existing control measures might cast doubts on the benefits of removing such measures, and thus be less likely to perceive that the LWV policy would greatly improve the economy and daily life, which in turn reduce the support. Notably, the large fifth-wave outbreak might imply that the existing control measures were ineffective and hence increase the level of support for the LWV policy in the near future.

The present study has implications for practice and research. Understanding the perceptions specific to a public health policy is important as it might influence compliance with the policy (15). This study reported the levels of perceptions specific to the LWV policy and their associations with the support for the LWV policy. The results might facilitate health communication between the government and the public and provide empirical evidence for policymakers and health authorities to develop more effective strategies amid the COVID-19 pandemic by modifying the identified perceptions. Although COVID-19 has become an endemic and almost all countries are adopting the LWV policy, the LWV policy is not a static one. When there are need to consider whether to exercise or relax the control measures (policy shift) in future pandemics, the findings of this study might inform future public health strategies and preparedness plans. This might happen sooner or later as new viruses keep emerging and evolving, including the new highly infectious COVID-19 strain (JN1). Based on the findings of this study, it seems that balancing public health protection with the minimization of adverse social, psychological, and economic impacts may be the key points to address when facing policy shifts in future pandemics.

It is a limitation of this study that the response rate was 52.5%, although it was comparable to that of other COVID-19-related telephone surveys in Hong Kong (34, 35). Other data collection methods were considered but deemed ineffective. First, household surveys do not necessarily increase the response rate. Furthermore, it was not a preferred mode during the pandemic when person-to-person interactions were minimized or avoided. Second, the response rates of online surveys and mailing surveys are questionable. Third, mobile phones were not included in this study as there was no available sampling frame and the older and less educated groups tended to be non-users. Like other telephone surveys (34, 35), the sampling frame of this study was based only on fixed-line telephones covering about 80% of all the households in Hong Kong as of May 2022. Notably, the age distribution of this study was comparable to that of the 2019 Hong Kong census (aged 18–30: 14.2% versus 12.4%; aged 31–60: 52.4% versus 53.3%; aged >60: 33.4% versus 34.2%) (36); the proportion of females was slightly overrepresented, although sex was not associated with support for the LWV policy in this study. It was also possible that the willingness to participate in the present study might be affected by the attitude towards the LWV policy. Those who were supportive of the LWV policy might be keener to express their attitude, or vice versa. Since both circumstances might occur, the two types of bias might offset each other.

There are other limitations of this study. Social desirability bias may exist, as the DCZ policy was officially endorsed by the Hong Kong government. Causal or temporal inferences were precluded by the cross-sectional design; the findings should be confirmed by longitudinal studies. Some items (e.g., perceived benefits and negative impacts of the LWV policy) were constructed for this study as validated scales were unavailable. Some scales consisted of single items. Other crucial factors of the LWV policy were not included in this study (e.g., perceived risk of infection). Although other countries may have similar concerns about the LWV policy, the level and factors might differ. Generalizations of the results thus should be made cautiously.

## 5 Conclusion

The present study observed that Hong Kong citizens were split in their support for the LWV policy in terms of the levels of both the support and perceived positive/negative impacts of the policy. About half of the public supported/strongly supported the LWV policy, showed concerns about causing many unnecessary deaths and the collapse of the local medical system, and did not believe that the policy would greatly improve the economy and daily life. The negative attitudes, together with perceived efficacy of the existing COVID-19 control measures and perceived harms of the Omicron variant, were negatively associated with the support for the LWV policy. These findings were reported in this study for the first time, as well as the significant mediation mechanisms involving the interrelationships between the above perceptions and the supportive attitude. These findings might facilitate health communication between health authorities and the public by identifying some modifiable perceptions to improve the acceptance and compliance with a public health policy. The results may also be applicable to future pandemics when similar control measures (e.g., social distancing) need to be exercised, relaxed, or installed.

## Data availability statement

The raw data supporting the conclusions of this article will be made available upon reasonable request by the corresponding authors.

## Ethics statement

The study was approved by the Survey and Behavioral Research Ethics Committee of the Chinese University of Hong Kong (No. SBRE-21-0555A). The studies were conducted in accordance with the local legislation and institutional requirements. The ethics committee/institutional review board waived the requirement of written informed consent for participation from the participants or the participants' legal guardians/next of kin because data was collected via a telephone survey where oral informed consent is required.

## Author contributions

JL: Conceptualization, Data curation, Funding acquisition, Methodology, Resources, Supervision, Validation, Writing – original draft, Writing – review & editing. JN: Investigation, Writing – review & editing. RG: Writing – review & editing. TK: Writing – review & editing. VF: Investigation, Writing – review & editing. ML: Data curation, Investigation, Writing – review & editing. YY: Data curation, Formal analysis, Methodology, Software, Writing – original draft, Writing – review & editing.
