# Supplementary material for: Level and factors of support for the Living with the Virus policy in a Chinese adult general population: a mediation analysis via positive and negative attitudes toward the policy
Source: Front Public Health. 2024 Jan 29;12:1286596. doi: 10.3389/fpubh.2024.1286596 (PMC10860402; doi:10.3389/fpubh.2024.1286596)
Supplement: Supplementary file 1 [file Table_1.DOCX]

Supplementary Material

Table S1 Correlation matrix among potential factors of support for the LWV policy

|  | 1 | 2 | 3 | 4 | 5 |
| --- | --- | --- | --- | --- | --- |
| 1. Perceived efficacy of the existing COVID-19 control measures | - |  |  |  |  |
| 2. Perceived physical harms of the Omicron variant | 0.24*** | - |  |  |  |
| **Perceived benefits of the LWV policy** |  |  |  |  |  |
| 3. Contribution to the control of the pandemic | −0.08 | −0.11* | - |  |  |
| 4. Great improvement in the economy and daily life | −0.20*** | −0.26*** | 0.42*** | - |  |
| **Perceived negative impacts of the LWV policy** |  |  |  |  |  |
| 5. Causing many unnecessary deaths | 0.18*** | 0.28*** | −0.41*** | −0.41*** | - |
| 6. Causing the collapse of the local medical system | 0.16*** | 0.23*** | −0.39*** | −0.36*** | 0.71*** |

Note. LWV = Living with the virus. *, *p*<0.05; ***, *p*<0.001.

Table S2 Testing the moderation effect of COVID-19 ever infection between cognitive factors and support for the LWV policy

|  | Main-effect-only | Main-effect + Interaction term |
| --- | --- | --- |
|  | ORa (95% CI) | ORa (95% CI) |
|  | *Model 1a* | *Model 2a* |
| Perceived efficacy of the existing COVID-19 control measures | 0.60 (0.44, 0.83)** | 0.67 (0.48, 0.94)* |
| COVID-19 ever infection | 2.11 (1.25, 3.55)** | 14.19 (0.85, 237.65) |
| Perceived efficacy of the existing COVID-19 control measures × COVID-19 ever infection |  | 0.57 (0.26, 1.28) |
| Δ −2LL | 2.014 | |
|  | *Model 1b* | *Model 2b* |
| Perceived physical harms of the Omicron variant | 0.77 (0.60, 0.99)* | 0.77 (0.58, 1.03) |
| COVID-19 ever infection | 1.99 (1.18, 3.35)** | 2.07 (0.32, 13.17) |
| Perceived physical harms of the Omicron variant × COVID-19 ever infection |  | 0.99 (0.56, 1.75) |
| Δ −2LL | 0.001 | |
|  | *Model 1c* | *Model 2c* |
| Contribution to the control of the pandemic | 2.33 (1.85, 2.94)*** | 2.51 (1.93, 3.26)*** |
| COVID-19 ever infection | 2.32 (1.34, 4.02)** | 7.66 (1.27, 46.40)* |
| Contribution to the control of the pandemic × COVID-19 ever infection |  | 0.66 (0.37, 1.19) |
| Δ −2LL | 1.779 | |
|  | *Model 1d* | *Model 2d* |
| Great improvement in the economy and daily life | 3.01 (2.26, 4.00)*** | 3.20 (2.32, 4.41)*** |
| COVID-19 ever infection | 1.72 (0.99, 3.01) | 5.82 (0.40, 83.75) |
| Great improvement in the economy and daily life × COVID-19 ever infection |  | 0.72 (0.36, 1.45) |
| Δ −2LL | 0.804 | |
|  | *Model 1e* | *Model 2e* |
| Causing many unnecessary deaths | 0.57 (0.47, 0.70)*** | 0.56 (0.45, 0.70)*** |
| COVID-19 ever infection | 2.06 (1.22, 3.48)** | 1.51 (0.25, 9.13) |
| Causing many unnecessary deaths × COVID-19 ever infection |  | 1.10 (0.65, 1.88) |
| Δ −2LL | 0.121 | |
|  | *Model 1f* | *Model 2f* |
| Causing the collapse of the local medical system | 0.62 (0.51, 0.76)*** | 0.61 (0.49, 0.75)*** |
| COVID-19 ever infection | 2.40 (1.42, 4.06)** | 1.37 (0.19, 9.72) |
| Causing the collapse of the local medical system × COVID-19 ever infection |  | 1.17 (0.69, 1.97) |
| Δ −2LL | 0.334 | |

Note. LWV = Living with the virus; ORa = Adjusted odds ratio; CI = Confidence interval; LL = Log likelihood. *, *p*<0.05; **, *p*<0.01; ***, *p*<0.001. The models were adjusted for socio-demographics, including sex, age, educational level, marital status, chronic disease status, and employment status.
